# Outcomes of subsyndromal delirium in ICU: a systematic review and meta-analysis

**DOI:** 10.1186/s13054-017-1765-3

**Published:** 2017-07-12

**Authors:** Rodrigo B. Serafim, Marcio Soares, Fernando A. Bozza, José R. Lapa e Silva, Felipe Dal-Pizzol, Maria Carolina Paulino, Pedro Povoa, Jorge I. F. Salluh

**Affiliations:** 1 Instituto D’Or de pesquisa e ensino, Rua Diniz Cordeiro, 30 Botafogo, Rio de Janeiro, RJ 22281-100 Brasil; 2 Hospital Copa D’Or, Rio de Janeiro, Brasil; 3 Hospital Universitário Clementino Fraga Filho/Instituto de Doenças do Tórax, Universidade Federal do Rio de Janeiro, Faculdade de Medicina, Av. Prof. Rodolpho Paulo Rocco, 255, Ilha do Fundão, 21941913 Rio de Janeiro, RJ Brasil; 4 Programa de pós-graduação em clinica médica, Universidade Federal do Rio de Janeiro, Rio de Janeiro, Brasil; 5 Instituto Nacional de Infectologia Evandro Chagas, FIOCRUZ, Rio de Janeiro, Brasil; 6 Unidade de Cuidados Intensivos Polivalente, Hospital de São Francisco Xavier, Centro Hospitalar de Lisboa Ocidental, Estrada do Forte do Alto do Duque, 1449-005 Lisboa, Portugal; 7 CEDOC, NOVA Medical School, Universidade Nova de Lisboa, Lisboa, Portugal; 8 Laboratório de patofisiologia experimental, Programa de graduação em ciências médicas, Universidade do Extremos Sul Catarinense, Avenida Universitária, 1105, C-postal: 3167, 88806-000 Criciuma, SC Brasil

**Keywords:** Subsyndromal delirium, Delirium, ICU, Critically ill

## Abstract

**Background:**

Subsyndromal delirium (SSD) is a frequent condition and has been commonly described as an intermediate stage between delirium and normal cognition. However, the true frequency of SSD and its impact on clinically relevant outcomes in the intensive care unit (ICU) remains unclear.

**Methods:**

We performed a systematic search in PubMed, Embase, CINAHL, Cochrane Library, and PsychINFO, with no language restrictions, up to 1 October 2016 to identify publications that evaluated SSD in ICU patients.

**Results:**

The six eligible studies were evaluated. SSD was present in 950 (36%) patients. Four studies evaluated only surgical patients. Four studies used the Intensive Care Delirium Screening Checklist (ICDSC) and two used the Confusion Assessment Method (CAM) score to diagnose SSD. The meta-analysis showed an increased hospital length of stay (LOS) in SSD patients (0.31, 0.12–0.51, *p* = 0.002; *I* ^2^ = 34%). Hospital mortality was described in two studies but it was not significant (hazard ratio 0.97, 0.61–1.55, *p* = 0.90 and 5% vs 9%, *p* = 0.05). The use of antipsychotics in SSD patients to prevent delirium was evaluated in two studies but it did not modify ICU LOS (6.5 (4–8) vs 7 (4–9) days, *p* = 0.66 and 2 (2–3) vs 3 (2–3) days, *p* = 0.517) or mortality (9 (26.5%) vs 7 (20.6%), *p* = 0.55).

**Conclusions:**

SSD occurs in one-third of the ICU patients and has limited impact on the outcomes. The current literature concerning SSD is composed of small-sample studies with methodological differences, impairing a clear conclusion about the association between SSD and progression to delirium or worse ICU clinical outcomes.

**Electronic supplementary material:**

The online version of this article (doi:10.1186/s13054-017-1765-3) contains supplementary material, which is available to authorized users.

## Background

Subsyndromal delirium (SSD) is a frequent condition characterized by a less severe cognitive impairment in comparison to delirium, in which some, but not all, diagnostic criteria for delirium are met [1]. However, to date there is no published consensus on the definitions for a subclinical form of delirium and SSD has been commonly reported as an intermediate stage between delirium and normal mental states [1]. The most frequently employed delirium screening tools consider the diagnosis of SSD when the Intensive Care Delirium Screening Checklist (ICDSC) score is 1–3 out of 8 [2] or when the Confusion Assessment Method (CAM) score is positive in two items out of four items [3].

The Diagnostic and Statistical Manual of Mental Disorders, 5th Edition (DSM-V), Neurocognitive Disorders Workgroup used the term “attenuated delirium syndrome” to describe a condition very similar to SSD but without specific diagnostic criteria and has been discussing whether SSD should be added as a subcategory of delirium in parallel with a new category, mild neurocognitive disorder. Of note, neither the DSM-IV-TR nor the DSM-V Workgroup determine nor distinguish whether subsyndromal presentations do or do not progress to delirium [4, 5].

Moreover, recent studies did not show a consistent association between SSD and increased mortality rates or clinical outcomes in intensive care unit (ICU) patients [6–8]. Nevertheless, despite limited available knowledge, intervention studies were recently performed aimed at the reduction of conversion from SSD to clinical delirium as a means to improve outcomes [9, 10]. We therefore conducted a systematic review of studies that evaluated SSD in the ICU. Our main objective was to produce quantitative estimates of the prevalence of SSD and to explore the association between SSD and clinically relevant outcomes of ICU patients such as the mortality, ICU and hospital length of stay (LOS), duration of mechanical ventilation (MV), and conversion to delirium.

## Methods

### Data sources and study selection

We conducted a systematic review and meta-analysis of prospective observational studies and clinical trials following the recommendations of the Meta-analysis Of Observational Studies in Epidemiology (MOOSE) group [11] and according to the recommendations of the Preferred Reporting Items for Systematic Reviews and Meta-Analyses (PRISMA) statement [12]. We searched the following data sources: Medline (1966 to 1 October 2016), Embase (1974 to 1 October 2016), CINAHL (1982 to 1 October 2016), the Cochrane Library (1 October 2016), and PsychINFO (1887 to 1 October 2016). The most recent search was performed on 1 October 2016. Reference lists of retrieved articles and of relevant review articles, as well as personal files, were hand searched. There was no language restriction. Search terms included: *subsyndromal delirium*, *subclinical delirium*, *delirium*, *agitation* which were cross-referenced with the terms *intensive care*, *intensive care unit*, *ICU*, *critical care*, *critical illness*, *critically ill*, *sepsis*, *acute respiratory distress syndrome*, *multiple organ system failure*, and *mechanical ventilation*. We considered the following criteria for study inclusion: 1) full-length reports published in peer-reviewed journals; 2) prospective observational cohorts or clinical trials of adult (>16 years old) patients admitted to the ICU; 3) use of validated screening or diagnostic instrument for delirium: CAM [13]; Confusion Assessment Method for the Intensive Care Unit (CAM-ICU) [3], ICDSC [2], DSM-IV TR [14], and DSM-V [5]; and 4) the relationship between SSD and at least one of the following outcomes reported: hospital and ICU LOS, MV duration, death in the ICU, conversion from subsyndromal delirium to delirium, or any post-hospital discharge outcomes. We excluded case studies or series and studies in which the majority of enrolled patients (or the largest subgroup) presented with the following conditions: primary central nervous system disorder (e.g., stroke, traumatic brain injury, central nervous system infections, brain tumors, recent intracranial surgery); underwent organ/tissue transplantation (patient subsets associated with pathophysiologically distinct forms of acute brain dysfunction); or experienced alcohol or substance withdrawal. Two investigators (JIFS and RBS) performed the study selection process including the initial search for the identification of references, the selection of potentially relevant titles for review of abstracts and, among these, those chosen for review of the full-length reports. All selections were decided by consensus.

### Data extraction and study quality assessment

Data extraction from the selected articles was independently performed by two authors (RBS and JIFS). The following data were recorded (when available): study characteristics (study location, period of enrollment, type of ICU, patient enrollment criteria, number of patients enrolled, methods used to identify delirium, duration of follow-up); patient characteristics (i.e., age, sex, premorbid cognitive and functional status, severity of illness scores, organ dysfunction scores and MV); and SSD prevalence and outcomes (i.e., conversion to delirium, death in the ICU and hospital, ICU and hospital LOS, and duration of MV).

To assess the methodological quality of the studies, we used the Newcastle–Ottawa Quality Assessment Scale (NOS) [15]. The scale evaluates three aspects of study methods: the selection of study groups (range 0–4), the comparability of groups (range 0–1), and the quality of outcome ascertainment (range 0–3). The total score ranges from 0 to 8, and an acceptable methodological design is reflected by a score >5.

### Analytical approach

We evaluated patient characteristics, prevalence of SSD and outcomes (conversion to delirium, mortality in the ICU and hospital, ICU and hospital LOS, and duration of MV) for patients with and without SSD. The main outcome of interest was mortality (ICU and hospital). The strength of the relationship between SSD and mortality was expressed as risk ratios (RRs) with 95% confidence intervals (CIs). We selected the risk ratio as a measure of effect for the binary outcome (death) since it is less prone to artificial inflation due to heterogeneity than risk difference. For continuous outcomes, we calculated the weighted standard mean difference (SMD) based on reported means or medians. We used the *I* ^2^ test to describe the proportion of the total variation in the study estimates that is due to heterogeneity in the meta-analysis. We performed all analyses using Review Manager version 5.3 [16].

## Results

### Search results and description of studies

**Fig. 1:**
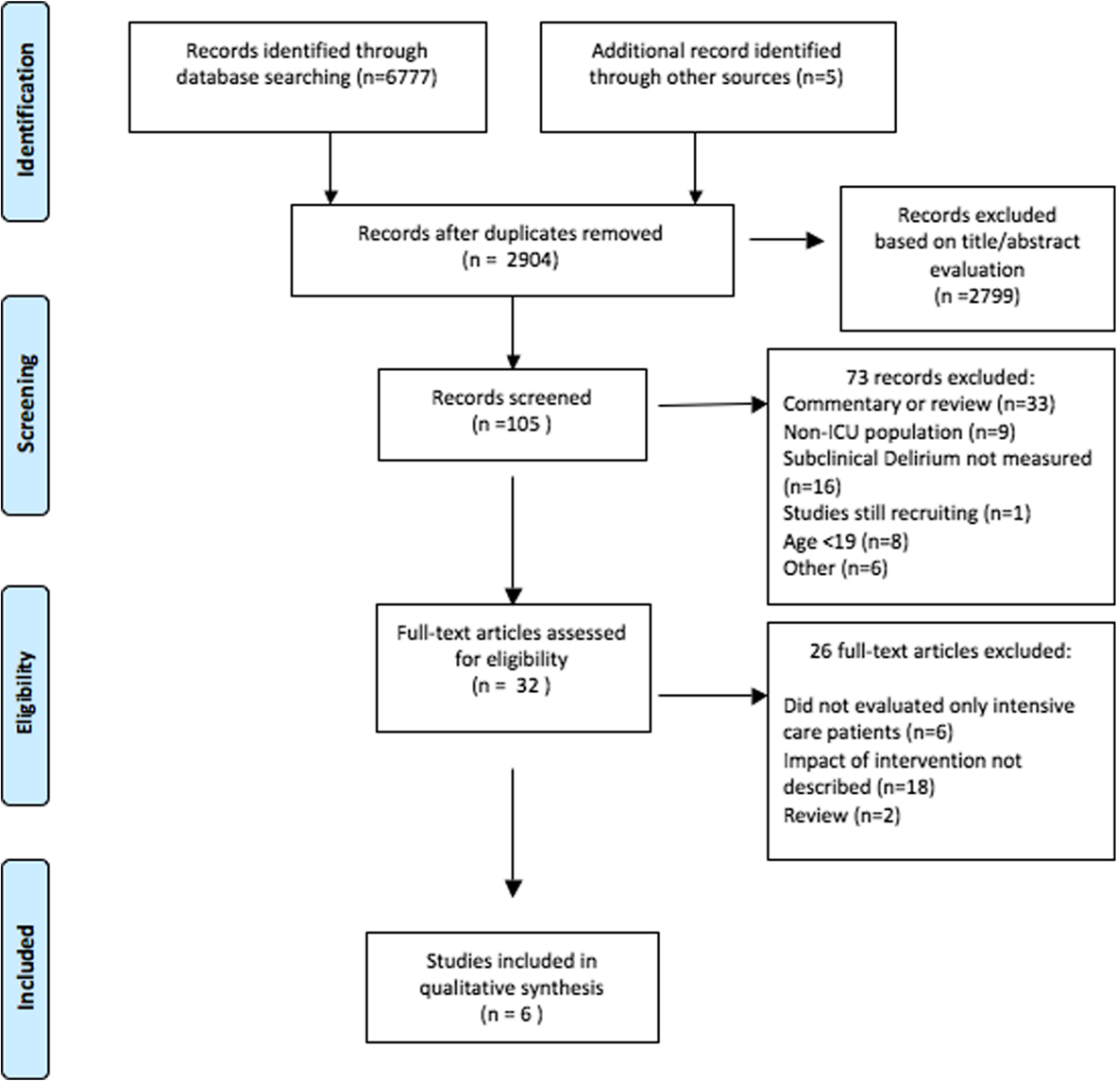
Subsyndromal delirium *flow diagram*. *ICU* intensive care unit

The initial search identified 6777 citations, and five studies were additionally identified as a result of reviewing the reference lists of others articles. Articles in duplicate were removed (*n* = 3878); most of duplicates occurred because PubMed citations were also included in the Embase library. After careful evaluation of the abstracts, 32 articles were retrieved and reviewed in detail. Disagreements (*n* = 2) between the two evaluators were solved by discussion and reaching a consensus. Finally, six studies met the inclusion criteria and were selected. Figure 1 depicts the flow diagram of the study search and selection process according to the PRISMA methodology.

Characteristics of the six studies selected are described in Table 1. Four studies [7, 8, 10, 17] exclusively evaluated critically ill surgical patients and the majority of patients (95%) had undergone elective surgery, one study evaluated a mixed population of critically ill patients [6], and one exclusively evaluated mechanically ventilated patients in the ICU [15]. The ICDSC was used for the diagnosis of delirium and SSD in four studies [6, 7, 9, 10]. The CAM was the instrument used in the two other studies [8, 17].

**Table 1:** Characteristics of the six studies which evaluated subsyndromal delirium

Reference	Patients enrolled, *n*	Type of patients	Delirium screening tool	Patients with SSD, *n* (%)	No. of patients with delirium, *n* (%)	Hospital LOS in SSD group, days (SD)	Hospital LOS in delirium group, days (SD)	Hospital LOS in non-delirium group, days (SD)	NOS
Al-Qadheeb et al., 2016 [9]	1358	Mechanically ventilated	ICDSC	481 (35%)	282 (37%)	NA	NA	NA	5
Li et al., 2015 [8]	38	Surgical	CAM	13 (34%)	7 (18%)	18.9 (7.5)	22.4 (13.9)^+^	14.2 (3.7)^+^	6
Breu et al., 2015 [7]	467	Cardiac surgical	ICDSC	158 (39%)	54 (12%)	9.0 (3.8)	11 (6)*	8.0 (2.0)*	5
Hakim et al., 2012 [10]	177	Cardiac surgery in the elderly	ICDSC	101 (57%)	NA	NA	NA	NA	5
Tan et al., 2008 [17]	53	Cardiac surgical	CAM	18 (34%)	12 (23%)	NA	NA	NA	5
Oiumet et al., 2007 [6]	537	Medical/surgical	ICDSC	179 (33%)	189 (35%)	40.9 (47)*^1^	36.4 (28.9)*^2^	31.6 (46.5)*^1,2^	7

Values are shown as means (SD) or *n* (%) as indicated

*p < 0.01, ^+^ *p* = 0.49

*CAM* Confusion Assessment Method, *ICDSC* Intensive Care Delirium Screening Checklist, *LOS* length of stay, *NA* not available, *NOS* Newcastle–Ottawa quality assessment scale, *SD* standard deviation, *SSD* subsyndromal delirium

### Quality assessment of studies

All studies were prospective, with two clinical trials [9, 10] and four observational studies [6–8, 17]. Two studies were randomized clinical trials primarily designed to evaluate the use of antipsychotic drugs to prevent the conversion of SSD to delirium [9, 10]. The selected studies were well designed and the NOS quality assessment demonstrated a low bias risk in most of them (Table 1) [15]. The funnel plot of the included studies in the meta-analysis was performed and suggested a low publication bias (Additional file 1).

### Patient characteristics

The main characteristics of those with no delirium, SSD, and delirium were well described in three observational studies [6–8]. In general, they had similar baseline characteristics in each group with some exceptions. In the study of Breu et al. [7], which evaluated patients who underwent cardiac surgeries, those with no delirium were younger (65.9 ± 10.3 vs 67.5 ± 9.9 vs 69.7 ± 11.7 years, *p* < 0.01) and had less duration of extracorporeal circulation (91.4 ± 34.0 vs 109.6 ± 49.6 vs 113.2 ± 44.7 min, *p* < 0.01), when compared with SSD and delirium patients, respectively. In the study of Li et al. [8], patients with no delirium had received fewer units in blood transfusion (1.3 ± 2.9 vs 3.3 ± 5.4 vs 15.7 ± 13.6 units, *p* < 0.01) and presented intraoperative hypotension for a small period of time (13.3 ± 13.5 vs 24.6 ± 31.9 vs 81.4 ± 76.5 min, *p* = 0.01) when compared with SSD and delirium patients, respectively. In the study by Ouimet et al. [6], patients with no delirium were younger (age 60 ± 15 vs 65 ± 14 vs 64 ± 15 years, *p* < 0.01), had a higher proportion of surgical admission diagnoses (62.1% vs 47.5% vs 48.7%, *p* = 0.01), and had the lowest Acute Physiology and Chronic Health Evaluation (APACHE) II scores at admission (12.9 ± 6.9 vs 16.7 ± 7.8 vs 18.6 ± 8.0, *p* < 0.01) when compared with SSD and delirium patients, respectively.

### Main clinical outcomes

SSD prevalence and hospital LOS were the most frequently reported outcomes (Table 1). Pooling all studies, a total of 2630 patients were evaluated; among them, SSD was identified in 950 (36%) patients.

Two studies evaluated the association of SSD with mortality [6, 7]. Ouimet et al. [6] reported an increased ICU mortality in the SSD group (10.6% vs 2.4%, *p* = 0.002) in comparison to patients with no delirium, but in a post-ICU follow-up and after adjusting for age, APACHE II score, and medication-induced coma, the mortality rate was similar in the SSD patients (hazard ratio 0.97 (0.61–1.55), *p* = 0.90) when compared to patients with no delirium. The study of Breu et al. [7] found that hospital mortality rates were comparable between SSD and patients with no delirium patients (4 (0.8-7.1) vs 5 (3.4-21.1), *p* = 0.05).

**Fig. 2:**
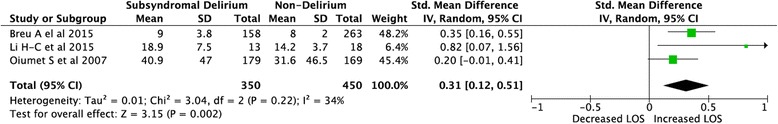
Forest plot comparing hospital length of stay (*LOS*) between SSD and non-delirium patients. Effects measure: risk ratio; analysis model: random effects; statistical method: *I* ^2^ heterogeneity. The ‘diamond’ at the *bottom* represents the 95% confidence interval (*CI*). *IV* initialization vector, *SD* standard deviation

Hospital LOS was described and compared between SSD, delirium, and non-delirium patients in only three studies [6–8]. Delirium was associated with longer hospital LOS when compared with non-delirium patients in all studies (Table 1). SSD was associated with longer hospital LOS when compared with non-delirium patients after meta-analysis performance (SMD 0.31 (95% CI 0.12–0.51), *p* = 0.002; *I* ^2^ = 34%) (Fig. 2).

Only two studies described the ICU LOS [6, 7] and one of them described a gradient with increasing ICU LOS in the comparison of those with non-delirium, SSD, and clinical delirium patients (respectively 2.5 ± 2.1, 5.2 ± 4.9, and 10.8 ± 11.3 days, *p* < 0.01) [6]. One study evaluated the MV duration and described a non-clinically relevant increase in weaning time (10.0 ± 8.0 vs 11.0 ± 10.75 h, *p* < 0.01) in SSD patients when compared to those without delirium [7].

### Effect of SSD treatment on its conversion to delirium

Two studies investigated the use of antipsychotic drugs to prevent the conversion from SSD to delirium [9, 10]. Al-Qadheeb et al. [9] described that the use of intravenous haloperidol 1 mg vs placebo every 6 h in SSD patients did not prevent conversion to delirium (12 (35.3%) vs 8 (23.5%), *p* = 0.29) or the time to first delirium occurrence (2 (2–3) vs 3 (2–4) days; *p* = 0.22), did not reduce delirium duration (2 (1–2) vs 3 (2–4) days, *p* = 0.261), ICU LOS (6.5 (4–8) vs 7 (4–9) days, *p* = 0.66), days on MV (4.5 (3–7) vs 5 (3–8), *p* = 0.79), or ICU mortality (9 (26.5%) vs 7 (20.6%), *p* = 0.55). In this study the sole observed difference was a reduced duration of agitation (0 (0–2) vs 2 (1–6) h, *p* = 0.008) in those receiving antipsychotics. Hakim et al. [10] described that the administration of risperidone (0.5 mg every 12 h) to elderly patients who experienced SSD after on-pump cardiac surgery was associated with a significantly lower prevalence of delirium by DSM criteria (7 (13.7%) vs 17 (34%), *p* = 0.031) and described fewer patients with ICDSC scores >3 (8 (15.7%) vs 19 (38%), *p* = 0.011) when compared with placebo. However, ICU and hospital LOS were comparable in both groups (2 (2–3) vs 3 (2–3) days, *p* = 0.517, and 6 (5–7) vs 6 (5–8) days, *p* = 0.056, respectively) as well as duration of clinical delirium (3 (2–4) vs 3 (3–4) days, *p* = 0.664).

## Discussion

In the present systematic review we synthesized the data on the prevalence of SSD in patients admitted to the ICU, as well as the association between SSD and delirium and clinical outcomes in critically ill patients. We identified six studies enrolling a total of 2630 patients. SSD was present in 950 (36%) patients. Despite marked variations between studies (from 33% to 57%) at least one in every three patients fulfilled the criteria for SSD, confirming the notion that it is a highly prevalent condition.

The SSD was not consistently associated with increased mortality or worse outcomes, as opposed to current data on delirium [18]; however, our meta-analysis found an increase in hospital stay (Fig. 2). Only one study evaluated the association of SSD with duration of MV and was not able to report a clinically relevant outcome [7], although the current literature supports the notion that delirium is independently associated with an increase in MV duration [18–20].

Studies that evaluate non-ICU patients have described the outcomes of SSD (i.e., cognitive decline, functional decline, increased hospital LOS, and increased rates of admission to long-term institutions and death) as being poor in older people [1, 21–23]. Cole et al. [1] in a recent systematic review of non-ICU older patients described that SSD had an elevated prevalence (23% (9–42%)), and is a clinically important condition that falls on a continuum between no symptoms and full delirium considering hospital LOS, post-discharge mortality, and functional decline [1]. Despite the apparent importance of SSD in non-ICU settings the increased mortality described above is not observed in the ICU population. Moreover, post-ICU discharge information about the role of SSD on long-term outcomes such as cognitive impairment or functional decline is not currently available.

There are important differences when we compare ICU and non-ICU patients regarding SSD that may explain the observed discrepancies in the outcomes. SSD may be a marker for underlying medical conditions not severe enough to cause full delirium in the non-ICU population where the cognitive trajectory and baseline severity of illness leads to a slowly increasing number of risk factors. In contrast, studies in ICU patients reported that at least 11 variables were considered to have moderate or strong evidence for contributing to delirium and they often occur simultaneously and are usually present in the first days following ICU admission [24, 25]. This high burden of non-modifiable risk factors present early at the onset of critical illness can contribute to the occurrence of delirium without a prodromal phase or SSD in the ICU. Moreover, as described by Patel et al. [26], the rapidly reversible sedation-related delirium showed fewer MV days (2.5 (1.6–2.8) vs 6.2 (3.7–12.0), *p* < 0.001), ICU days (4.5 (2.2–7.2) vs 13.1 (8.8–19.1), *p* = 0.001), and hospital days (6.7 (3.8–16.4) vs 25.4 (13.6–29.6), *p* < 0.001) than persistent delirium. Those patients with rapidly reversible sedation-related delirium had lower hospital mortality in comparison with persistent delirium (0% vs 36%, *p* = 0.001), which was similar to subjects with no delirium [26]. This may also indicate that the occurrence of SSD (a condition of lower severity as compared with rapidly reversible delirium) may not be sufficient to generate worse outcomes or may be a transient condition from a worse neurologic state (such as coma or delirium) before re-establishing normal cognition.

The conversion of mental status or percentage of transition from SSD to delirium was only evaluated in two small-sample clinical trials to describe the effect of antipsychotics in preventing the conversion from SSD to delirium [9, 10]. In the study of Al-Qadheeb et al., 1358 patients were evaluated but only 68 patients were classified as SSD and received intervention [9].

Therefore, as the course of delirium can be heterogeneous and unpredictable it is unclear if the presence of SSD during the trajectories of delirium or even subsequent development of residual SSD after recovery can be implicated in negative outcomes.

From the two studies that evaluated the use of antipsychotic drugs in SSD patients, only one study could show a reduced conversion from SSD to delirium [10]. Moreover, none of these studies demonstrated any other positive impact in relevant outcomes such as ICU and hospital LOS or mortality. These findings appear to be in concordance with the current literature on delirium which provides no conclusive evidence that pharmacologic treatment of ICU delirium modifies clinically relevant outcomes other than agitation [27].

Finally, the absence of a formal definition of SSD contributes to the heterogeneity between studies and may partially explain the conflicting results described. Some researchers have defined SSD as having at least two or more a priori selected core symptoms [21, 22], whereas others have specifically focused on attention and cognitive impairments [28] or have used specific cut-off points on diagnostic scales for delirium, such as in the ICDSC [6]. The ICDSC scale was developed to diagnose and graduate the delirium symptoms [2] and it is more in line with the proposed diagnosis of SSD. The CAM was used by authors to diagnosis SSD in ICU [8, 17] and non-ICU settings [1], but considers only the presence of some symptoms not fulfilling the criteria for delirium. At this point, the CAM forces a dichotomization in diagnosis of mental status and it is not clear if the items of the CAM represent the same relevance in cognitive dysfunction or the same impact in the outcomes. Recently, two new delirium rating scales were described, called CAM-S [29] and the CAM-ICU-7 [30]. They were derived from CAM and CAM-ICU, respectively, and were able to provide a graded scale for delirium severity assessment. This is in contrast to ICDSC, which plateaus at the threshold of clinical delirium and does not provide further predictive discrimination. However, the data available were limited to delirious patients only. For future studies it will be necessary to clarify assessment of SSD using the new scales.

The clinical entity and the implications of SSD are not fully understood or precisely defined. It remains unclear whether SSD represents an early stage of manifest full delirium, an independent diagnosis, or simply a description for an array of symptoms with no major clinical consequence.

The present study has some limitations and several of them are actually related to the aforementioned absence of a clear definition of SSD. First, we consider that, despite being a major drawback, the diagnoses of SSD for patients in the present study are those being used by clinicians in their practice as well as investigators in clinical studies. We believe that differences in the design, definitions, and tools to diagnose delirium and available data on outcomes and disease severity as well as substantially diverse patient populations could account for the substantial differences in prevalence and mortality. Second, none of the studies evaluated SSD using the CAM-ICU. ICU providers do not typically use the CAM, considering that many patients are not able to communicate or it takes too long and requires special training, which could contribute to an underdiagnosis of delirium. Third, the assessment of publication bias through the funnel plot analysis was impaired due to the small sample size; the power of the test is too low to distinguish chance from real asymmetry. Fourth, we did not conduct a grey literature search which might contribute to an overestimation of the size effect in small trials. Finally, since data on long-term outcomes are not available in the current literature, this relevant aspect of SSD could not be evaluated.

## Conclusion

SSD is a frequent condition that is present in nearly one-third of ICU patients. The current literature concerning SSD is composed of small-sample studies with huge methodological differences between them, impairing a clear conclusion on the association between SSD with progression to delirium or its impact on clinical outcomes in the ICU. Considering the present results, SSD has limited impact on the outcomes, and future studies should focus on the evaluation of larger populations of critically ill patients employing standardized definitions, thorough risk assessment, and clinically relevant outcome measures for a better understanding of the relevance of SSD in ICU patients as well as its treatment.

## Additional file

Additional file 1: Funnel plot of articles in the meta-analysis comparing hospital length of stay between subsyndromal delirium and non-delirium patients. (TIFF 22 kb)

## Abbreviations

APACHE: Acute Physiology and Chronic Health Evaluation
CAM: Confusion Assessment Method
CAM-ICU: Confusion Assessment Method for the intensive care unit
CI: Confidence interval
DSM: Diagnostic and Statistical Manual of Mental Disorders
ICDSC: Intensive Care Delirium Screening Checklist
ICU: Intensive care unit
LOS: Length of stay
MV: Mechanical ventilation
RR: Risk ratio
SMD: Standard mean difference
SSD: Subsyndromal delirium
