# Supplementary figures and images for: Outcomes of subsyndromal delirium in ICU: a systematic review and meta-analysis
Source: Crit Care. 2017 Jul 12;21:179. doi: 10.1186/s13054-017-1765-3 (PMC5506578; doi:10.1186/s13054-017-1765-3)

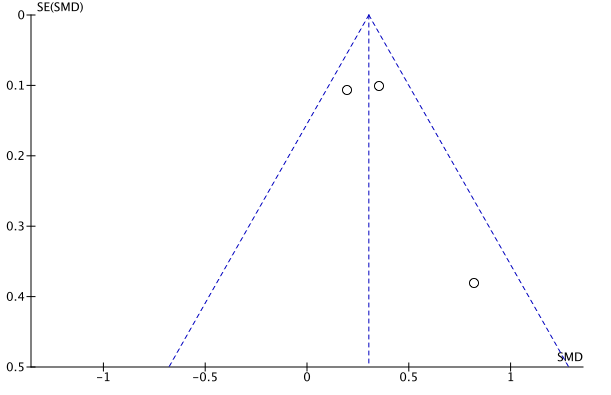

Supplement: Additional file 1: — Funnel plot of articles in the meta-analysis comparing hospital length of stay between subsyndromal delirium and non-delirium patients. (TIFF 22 kb) [file 13054_2017_1765_MOESM1_ESM.tiff]
